# Vanadium dioxide–zinc oxide stacked photocathodes for photo-rechargeable zinc-ion batteries†

**DOI:** 10.1039/d1ta07572a

**Published:** 2021-10-07

**Authors:** Buddha Deka Boruah, Michael De Volder

**Affiliations:** Department of Engineering, University of Cambridge Cambridge CB3 0FS UK bd411@cam.ac.uk mfld2@cam.ac.uk

## Abstract

The development of batteries that can be recharged directly by light, without the need for external solar cells or external power supplies, has recently gained interest for powering off-grid devices. Vanadium dioxide (VO_2_) has been studied as a promising photocathode for zinc-ion batteries because it can both store energy and harvest light. However, the efficiency of the photocharging process depends on electrode structure and charge transport layers. In this work, we report photocathodes using zinc oxide as an electron transport and hole blocking layer on top of which we synthesise VO_2_. The improved interface and charge separation in these photocathodes offer an improvement in photo-conversion efficiency from ∼0.18 to ∼0.51% compared to previous work on mixed VO_2_ photocathodes. In addition, a good capacity retention of ∼73% was observed after 500 cycles. The proposed stacked photocathodes reduce the battery light charging time by 3-fold and are therefore an important step towards making this technology more viable.

## Abstract 2

Layered zinc oxide–vanadium dioxide photocathodes for high efficiency zinc-ion photo-batteries.

## Graphical abstract

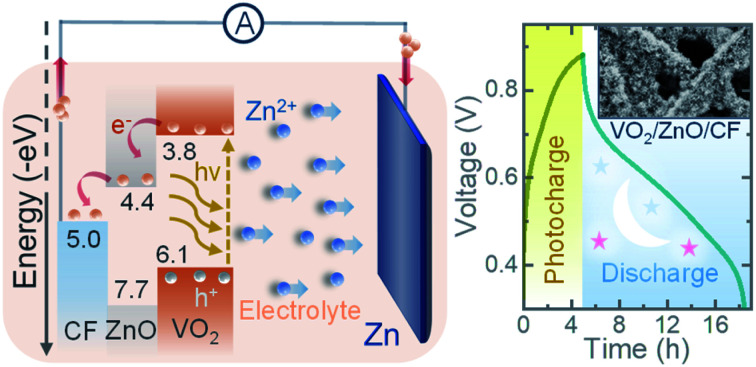

## Introduction

Batteries that can be recharged directly by light without the need for solar cells (photo-batteries) have been proposed more than 40 years ago,^1^ but have recently gained renewed attention due increased interest in renewable energy storage and off-grid sensors and internet of things (IoT) devices as well as developments in materials and tools in the battery and solar cell communities. One branch of photo-battery research is focusing on the development of battery materials that can simultaneously harvest solar energy and store it electrochemically. Compared to classic systems where solar cells and batteries are separate devices, photo-batteries offer a more compact design without the need for external electronics to match the output of the energy solar cell to the battery. Therefore, a range of different materials have been considered for photo-batteries, including, for instance, halide perovskites,^2,3^ organic molecules,^4^ vanadium pentoxide,^5,6^ germanium selenide,^7^ and titanium dioxide.^8^ Most photoelectrodes are fabricated by physically mixing of electrode materials with conductive additives, charge transfer materials and binders followed by casting on a collector electrode. While these processes are easy to scale up, random mixtures of photocathode materials with a charge transfer material and non-conducting binder may suffer from poor separation and transport of photocharges, resulting in limited energy conversion efficiencies.

Here, we show how the photocharge conversion efficiency of a vanadium dioxide (VO_2_) photocathode for zinc (Zn)-ion photo-batteries can be improved from ∼0.18% (ref. 9) to ∼0.51% (at 455 nm illumination) using an electrode design with improved interfaces and charge transfer materials. More specifically, in this work, we first coated a carbon fibre (CF) current collector with zinc oxide (ZnO) as a hole blocking and electron transport layer, followed by a direct growth of VO_2_ material. Finally, in this work we focus on Zn-ion batteries rather than lithium (Li)-ion batteries for a number of different reasons. First, Zn-ion batteries are using Zn metal anodes by default, which are more stable during plating and stripping than Li anodes and therefore half-cell and full cell tests are by definition the same for these batteries, which makes the use of cathodes such as VO_2_ that do not contain any Zn during synthesis viable. Second, Zn-ion batteries are known to be extremely cost-effective^10^ and therefore suitable for powering off-grid communities and fighting energy poverty. Finally, Zn-ion batteries oftentimes rely on aqueous electrolytes, which may be safer to operate at the high temperatures (>65 °C) solar panels can reach during operation.^11^

**Fig. 1:**
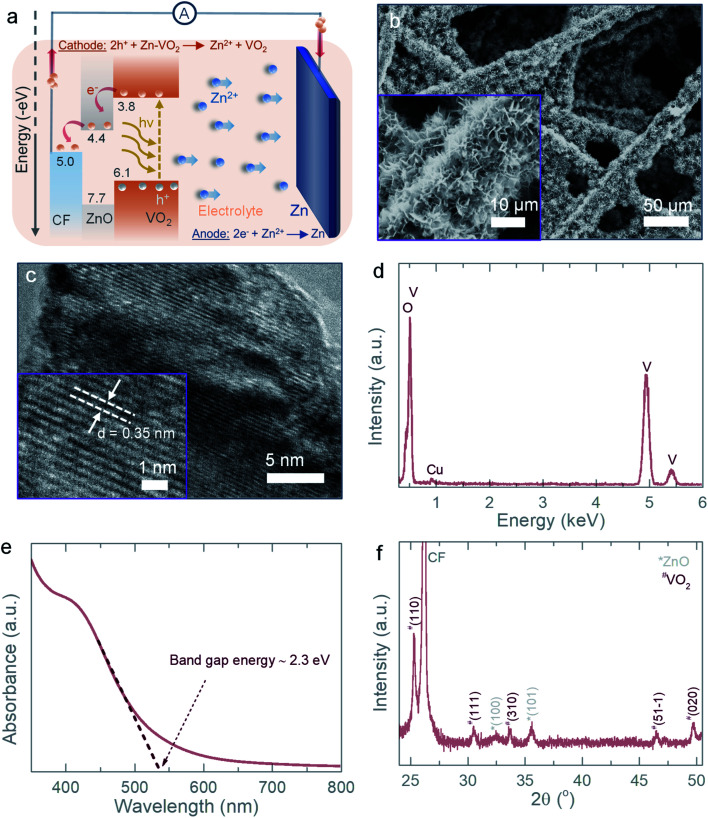
(a) Schematic illustration of photocharging mechanism of the proposed VO_2_/ZnO Zn-ion photo-battery. (b) SEM images of the VO_2_/ZnO photocathode. (c and d) High resolution-TEM image and the respective EDS spectrum of the used VO_2_. (e) UV-Vis absorption spectrum of VO_2_. (f) XRD pattern of the photocathode.

The photocharging mechanism of the proposed ZnO/VO_2_ Zn-ion photo-battery is depicted in Fig. 1a, where electrons are photo-exited to the conduction band of VO_2_ and then transported to CF through a ZnO layer, which is also used to blocks holes. This combined action of electron extraction and blocking holes in VO_2_ leads to photocharging (see further). Note that the ZnO layer is used for charge transport whereas VO_2_ is used for energy storage. Fig. 1b shows a scanning electron microscopy (SEM) image of a ZnO/VO_2_ electrode (SEM images of CF before and after ZnO coating are provided in the ESI, Fig. S1†). Various cathode materials including vanadium oxides, manganese oxides, and molybdenum disulfide, have widely been explored for Zn-ion batteries.^12^ Vanadium-based cathode materials offer a good charge storage capacity and reversible insertion/extraction of Zn ions with fast kinetics.^12^ VO_2_ is selected here because of its band gap energy in the visible light spectrum and fast charge–discharge kinetics. The latter is due to its frameworks structure with relatively large tunnels of ∼0.82 nm^2^ along *b*-axis and ∼0.5 nm^2^ along *c*-axis that enable insertion/extraction of Zn ions.^13^ Further, it has a high theoretical capacity and hence seem to be an attractive cathode material for Zn-ion battery applications.^14,15^Fig. 1c shows a high-resolution transmission electron microscopy (TEM) image of the VO_2_ that confirms an interplanar spacing of ∼0.35 nm (inset) corresponds to (110) planes of the monoclinic VO_2_ (B). Fig. 1d shows the corresponding energy dispersive X-ray spectroscopy (EDS) spectrum of the TEM image. The UV-Vis absorption spectrum of VO_2_ (Fig. 1e) is used to calculate the band gap energy of ∼2.3 eV in agreement with literature.^9,16^ Finally, X-ray diffraction (XRD) patterns in Fig. 1f confirm the phase of our material is monoclinic VO_2_ (B).

**Fig. 2:**
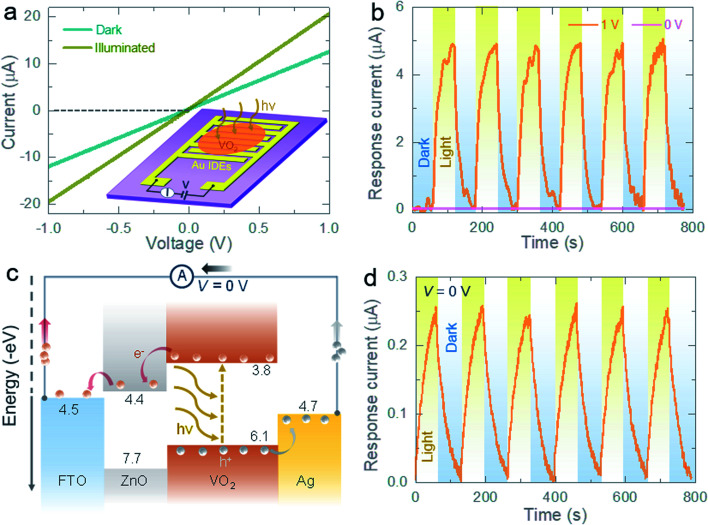
Electrical photoresponse measurements. (a) Current–voltage curves of a planar Au–VO_2_–Au IDEs photodetector in dark and illuminated (*λ* ∼455 nm) conditions. Inset shows schematic illustration of the IDEs photodetector. (b) Current–time curves of the Au–VO_2_–Au photodetector under alternating dark and illuminated conditions in absence (*V* = 0 V) and presence (*V* = 1 V) of an external bias voltage. (c and d) Energy band diagram of a stacked FTO/ZnO/VO_2_/Ag photodetector and the response current–time profile in alternating dark and light conditions in absence of a bias voltage (*V* = 0 V).

Before building photo-batteries, the photosensitivity and photo-charge separation/transportation between VO_2_ and ZnO are studied in photodetectors. Fig. 2a shows the current–voltage curves in dark and light of a planar gold (Au)–VO_2_–Au type metal–semiconductor-metal interdigitated electrodes (IDEs) photodetector (inset illustrates schematic representation of the IDEs photodetector whereas the digital photograph is included in Fig. S2a, ESI†), where, VO_2_ was drop casted on Au IDEs (see Experimental section, ESI†). As expected, the current response of the photodetector increases under illumination (*λ* ∼ 455 nm) due to photocharge carriers created in VO_2_. Fig. 2b shows the photocurrent generated under a bias voltage of 1 V (response current = *I*_ph_ − *I*_dark_; where *I*_dark_ and *I*_ph_ are the currents in dark and light conditions). In Fig. 2a and b, no current is observed at 0 V bias since there is no charge separation mechanism in a pure VO_2_ electrode. On the other hand, a fluorine doped tin oxide (FTO)/ZnO/VO_2_/silver (Ag) stacked photodetector is expected to create a photocurrent, even without a bias voltage as illustrated by the charge separation process in Fig. 2c (a digital photograph of the detector is provided in the ESI, see Fig. S2b†). The photocurrent generation without external bias voltage under light is confirmed in Fig. 2d, which implies that the proposed ZnO/VO_2_ electrodes are capable of separating and transporting of photocharges as required for a photo-battery.

**Fig. 3:**
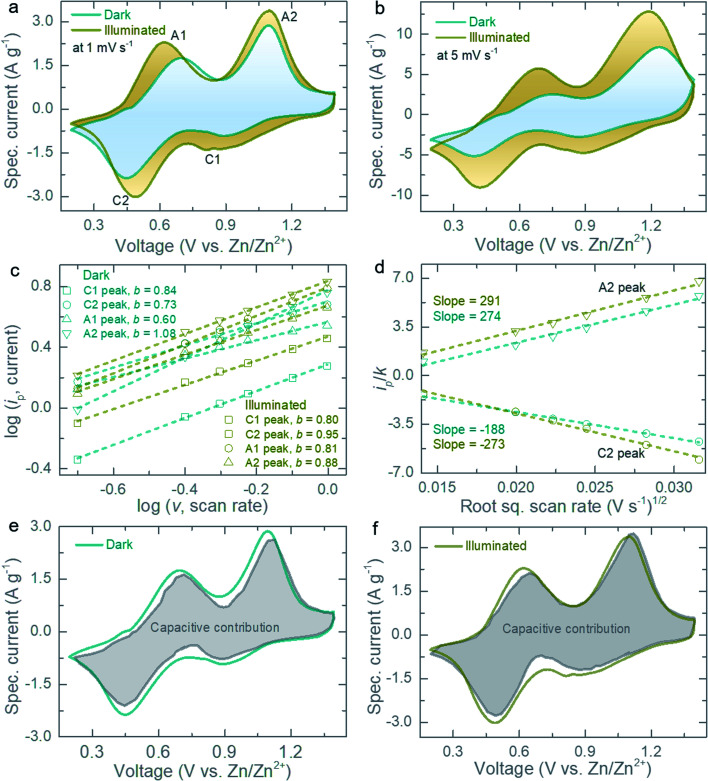
CV curves at (a) 1 mV s^−1^ and (b) 5 mV s^−1^ in dark and illuminated (*λ* ∼455 nm and intensity ∼12 mW cm^−2^) conditions. (c) Determination of *b*-values for cathodic (C1 and C2) and anodic (A1 and A2) peaks in dark and illuminated (*λ* ∼455 nm and intensity ∼12 mW cm^−2^) conditions. (d) Estimation of Zn ion diffusion constant improvements for major cathodic (C2) and anodic (A2) peaks under illumination. (e and f) Quantification of capacitive contribution to charge storage in dark and illuminated (*λ* ∼ 455 nm and intensity ∼12 mW cm^−2^) conditions at 1 mV s^−1^.

Next, electrochemical tests are carried out in optical coin cells (CR2450 with a ∼8 mm diameter optical, see Experimental section, Fig. S3†). The photocathodes are tested against Zn anode in aqueous 3 M Zn(CF_3_SO_3_)_2_ electrolyte in dark and illuminated conditions (*λ* ∼ 455 nm, ∼12 mW cm^−2^ unless stated otherwise). First, cyclic voltammograms (CV) scans are recorded at scan rates of 0.2 mV s^−1^ to 10 mV s^−1^ in dark and illuminated conditions (voltage window 0.2–1.4 V). The CV curves at 1 mV s^−1^ (Fig. 3a) show pairs of cathodic peaks (assigned as C1 and C2) and anodic peaks (A1 and A2) as expected from the multi-step intercalation and de-intercalation of Zn^2+^ into VO_2_.^13–15^ Light illumination increases the peak currents as expected from the photodetector experiments discussed above, with ∼28% and ∼72% enhancements in the CV area at 1 mV s^−1^ and 5 mV s^−1^ respectively (Fig. 3a and b). Additional CV curves at different scans (0.2–10 mV s^−1^) in dark and light are provided in the ESI (Fig. S4†). The CV curves show an increase in current under illumination over the entire voltage window, which suggests that VO_2_ maintains a bandgap independent of the states of charge (SoC) used here, which is consistent with previous work.^9^ We have verified this by carrying out *ex situ* UV-Vis analysis of electrodes at different SoC and calculating the bandgap (see Fig. S5 and Table S1†).

To quantify the capacity contributions from capacitive-controlled and diffusion-controlled processes in light and dark conditions, we correlate the peak current (*i*_p_) of the CV curves with scan rate (*v*) following the power–law relation, *i*_p_ = *av*^*b*^ or log(*i*_*p*_) = log(*a*) + *b* × log(*v*); where *a* and *b* are adjustable parameters. If *b* ≈ 0.5, the electrochemical process indicates a diffusion-controlled, whereas if *b* ≈ 1.0 the process is mainly dominated by capacitive-controlled.^17^ The calculated *b*-values for C1, C2, A1 and A2 are 0.84, 0.73, 0.60 and 1.0, respectively in dark conditions (Fig. 3c), which suggests overall capacity contribution from both capacitive and diffusive processes. Under illumination, capacitive contributions increase with *b*-values of 0.80, 0.95, 0.81 and 0.88 for C1, C2, A1 and A2 peaks respectively. Moreover, the peak current of CVs for C2 and A2 peaks can be related to the Zn ion diffusion constant (*D*) as follows,^18^*i*_p_ = 0.4463*F*(*F*/*RT*)^1/2^*ACD*^1/2^*v*^1/2^ = *KD*^1/2^*v*^1/2^; where *F*, *A* and *C*, represent Faraday constant, electrode area and initial concentration, respectively. If we assume *A* is not influenced by illumination, then *K* = 0.4463*F*(*F*/*RT*)^1/2^*AC* is a constant in dark and illumination conditions. Based on the slope of *v*^1/2^ plotted against *i*_p_/*K* (Fig. 3d and S6†), the calculated diffusion constant enhancements for major cathodic peak (C2)/anodic peak (A2) are ∼45%/∼6.2% (at scan rate range of 0.2–1.0 mV s^−1^) and ∼126%/∼150% (at 2–10 mV s^−1^) under illumination as compared to that of dark condition. In addition, the charge storage process can be evaluated into capacitive-controlled (*k*_1_*v*) and diffusion-controlled (*k*_2_*v*^1/2^) components and hence, at a fixed voltage of CVs the current can be expressed as,^19^*i*(*V*) = *k*_1_*v* + *k*_2_*v*^1/2^ or *i*(*V*)/*v*^1/2^ = *k*_1_*v*^1/2^ + *k*_2_. The calculated capacitive contributions are ∼87% for dark (Fig. 3e) and ∼93% for light (Fig. 3f) conditions (scan rate of 1 mV s^−1^).

**Fig. 4:**
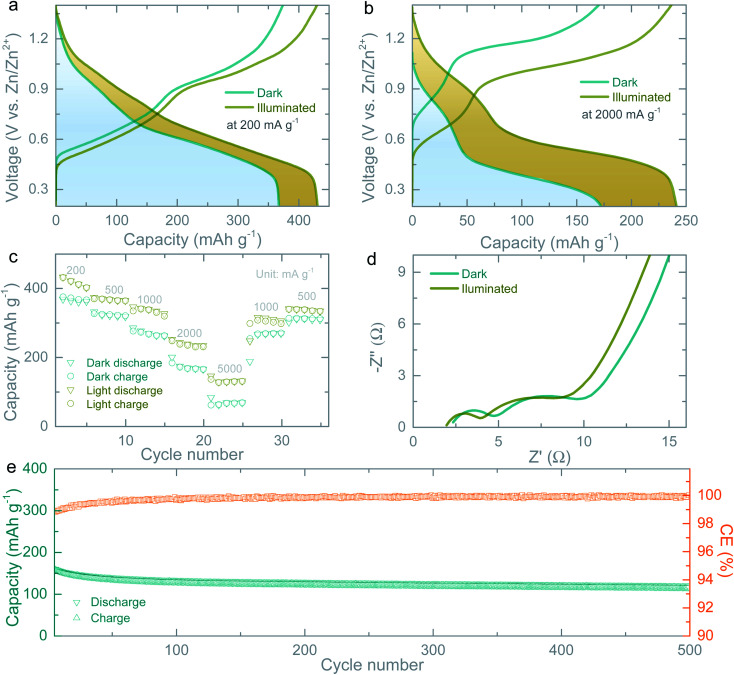
(a and b) GDC curves of the photo-batteries at 200 mA g^−1^ and 2000 mA g^−1^ both in dark and illuminated conditions (*λ* ∼ 455 nm and intensity ∼12 mW cm^−2^) after one formation cycle. (c) Rate capacity measurements in dark and illuminated (*λ* ∼ 455 nm and intensity ∼12 mW cm^−2^) conditions. (d) Nyquist plots of the photo-battery recorded after the 2^nd^ galvanostatic discharge cycle to 0.7 V in dark and illuminated (*λ* ∼455 nm and intensity ∼12 mW cm^−2^) states. (e) Cycling stability test of the photo-battery acquired at 1000 mA g^−1^ in dark condition with a capacity retention of ∼73% after 500 cycles which is estimated based on the after five formation cycles (Fig. S7†).

Next, we measure galvanostatic discharge–charge (GDC) curves at current densities of 100 mA g^−1^ to 5000 mA h g^−1^ in dark and illuminated conditions. As expected from the CVs curves, under illumination, the capacity of the photo-batteries increases from 367 mA h g^−1^ to 432 mA h g^−1^ at 200 mA g^−1^ (Fig. 4a) and from 172 mA h g^−1^ to 242 mA h g^−1^ at 2000 mA g^−1^ (Fig. 4b). The rate test results (Fig. 4c) further confirm the increase in capacity of the photo-batteries under illumination even at high specific current densities of 5000 mA g^−1^. The increase in the capacities of the photo-batteries under illumination as compared to that of dark condition are due to photocharges which are generated continuously under illumination. In addition to study the charge transfer characteristics of the photo-battery in dark and illuminated conditions, we measure Electrochemical Impedance Spectroscopy (EIS) which are recorded after the 2^nd^ galvanostatic discharge cycle to 0.7 V in light and dark conditions. As shown in Fig. 4d, the charge transfer resistance decreases from ∼10 Ω to ∼8.5 Ω and equivalent series resistance from ∼2.36 Ω to ∼1.98 Ω under illumination suggesting that light illumination not only improve the capacities but also decreases the impedance of the cells.^20,21^ Finally, long-term cycling test of the photo-battery in dark conditions shows a capacity retention of ∼73% after 500 cycles and a ∼98.8% coulombic efficiency (CE) after the five formation cycles (see Fig. 4e and S7†).

**Fig. 5:**
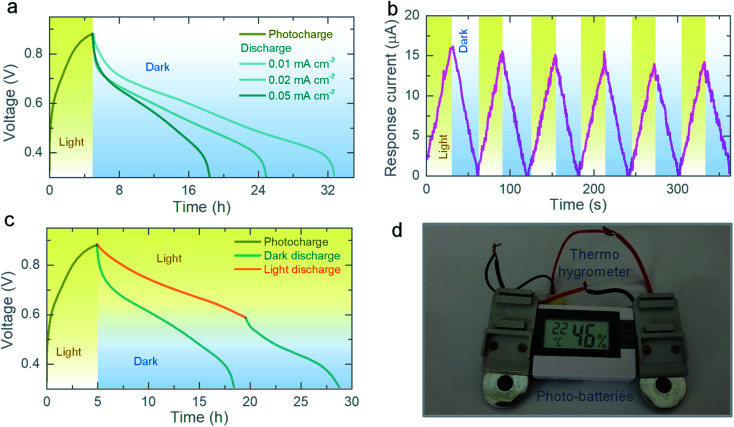
(a) Photocharge (*λ* ∼ 455 nm and intensity ∼12 mW cm^−2^) of the photo-battery and galvanostatic discharges at different specific currents. (b) Chronoamperometry test of the photo-battery under alternating dark and illuminated (*λ* ∼ 455 nm, intensity ∼12 mW cm^−2^) conditions at 0 V applied voltage, showing increase in the response current under light. (c) Photocharge (*λ* ∼ 455 nm and intensity ∼12 mW cm^−2^) and galvanostatic discharge in dark and illuminated conditions. (d) Digital photograph showing a 1.5 V Thermo-hygrometer powered by two photocharged photo-batteries.

To study the photo-charging process, we discharge the photo-batteries galvanostatically and then photocharge by light only without applying an external current. As shown in Fig. 5a, the output voltage of the photo-battery increases to ∼880 mV when illuminated for 5 h, and we subsequently discharge the cell at different specific currents. Under illumination (Fig. 1a), the photoexcited electrons are transported from the photocathode to the Zn anode through the external circuit and will accumulate in the Zn anode, where we they can reduce Zn ions to Zn metal (Zn^2+^ + 2e^−^ → Zn). The photoexcited holes on the other hand could increase the oxidation state of vanadium, which helps driving the deintercalation of Zn ions (Zn−VO_2_ + 2h^+^ → Zn^2+^ + VO_2_). We believe that the combination of these reactions at the anode and cathode drive the photo-charging process. Chronoamperometry tests in absence of external voltage (*V* = 0 V) shows an increasing response current under light, confirming transport of photoexcited charges through the cell along with Zn ions (see Fig. 5b). Moreover, when a cell is discharged under illumination, the photo-batteries discharge slower than in dark conditions because they are photocharged during the discharge process (see Fig. 5c).

Finally, we calculate the photo-conversion efficiency (*η* = *E*_out_/*E*_in_ × 100%, with *E*_out_ and *E*_in_ the discharge energy and accumulated light energy respectively). In our photo-batteries, *η* ∼ 0.51% (455 nm illumination), which is nearly 2.8 times higher than that of our previously reported VO_2_ mixed with rGO and PVDF photocathodes (*η* ∼ 0.18%), which is confirmed by a ∼ three time faster photo-charging time (see Table S2†).^9^ We expect that this increase in performance is due to an improvement in charge separation using ZnO as well as an improvement in the interface by synthesizing ZnO and VO_2_ directly on top of each other, rather than using physical mixtures of active material with charge transfer material and non-conducting binder. The efficiency achieved with VO_2_ is lower than a recent publications using V_2_O_5_,^5,6^ however, the capacity of VO_2_ reported in this work is more than two times higher than the previously reported V_2_O_5_ based photo-batteries. Further, our photo-batteries can be recharged by illuminating 1 sun as shown in Fig. S8.† In addition, we powered a commercial sensor (a 1.5 V Digital Thermo-Hygrometer TFA, MPN: 30.5005) by photo-batteries charged only by light as shown in Fig. 5d.

In conclusion, we demonstrate a Zn-ion battery that can be recharged directly by light, without using any external power source. This is achieved by using VO_2_ as both the material that stores Zn ions and photo-generates electron–hole pairs. Using photodetectors, we demonstrate that ZnO is a suitable electron transport and hole blocking layer for VO_2_, and we developed a process where ZnO is coated on the current collector first and VO_2_ is synthesised directly on the ZnO layer. This improved charge separation and the interface between active materials, resulting in a 2.8 times higher photo-conversion efficiency compared to materials where the VO_2_ is physically mixed with an electron transport material. Further, these binder free photocathodes attained capacities of ∼367 mA h g^−1^ in dark and 432 mA h g^−1^ in light at 200 mA g^−1^. Finally, we observed a capacity retention of ∼73% after 500 cycles.

## Conflicts of interest

The authors declare no competing financial interest.

## Supplementary Material

TA-009-D1TA07572A-s001
