# Supplementary material for: Vanadium dioxide–zinc oxide stacked photocathodes for photo-rechargeable zinc-ion batteries
Source: J Mater Chem A Mater. 2021 Oct 7;9(40):23199–205. doi: 10.1039/d1ta07572a (PMC8525630; doi:10.1039/d1ta07572a)
Supplement: TA-009-D1TA07572A-s001 [file TA-009-D1TA07572A-s001.pdf]

Electronic Supplementary Material (ESI) for Journal of Materials Chemistry A.
This journal is © The Royal Society of Chemistry 2021

Supporting Information

for

Vanadium Dioxide-Zinc Oxide Stacked Photocathodes for Photo-Rechargeable Zinc-Ion Batteries

Buddha Deka Boruah,[1,*] and Michael De Volder[1,*]

[1]Department of Engineering, University of Cambridge, Cambridge CB3 0FS, UK

*Corresponding Authors: Dr. Buddha Deka Boruah, E-mail: bd411@cam.ac.uk

Prof. Michael De Volder, E-mail: mfld2@cam.ac.uk

Experimental Section

Photocathode Preparation: ZnO solution was prepared by dissolving zinc acetate dehydrate (100 mg) in N,N-Dimethylformamid (5 mL). Next, the CF (Sigracet GDL 39 AA carbon graphite paper, SGL Carbon) current collectors were dipped into the solution followed by drying at 120 ^{o}C. This process was repeated for few times to get uniform coating, and finally dried the samples at 320 ^{o}C.

The VO_2 growth solution was prepared by dissolving ammonium metavanadate (2 g) and oxalic acid (3.75 g) into deionized water (120 mL) and then continued stirring for 12 h. Next, the solution was transferred into an autoclave reactor with the ZnO coated CF current collectors and then maintained at 180 °C for 12 h. Finally, the samples were collected and washed with deionized water and ethanol. As obtained samples were cut into specific dimensions for electrochemical testing.

Characterization: The morphological analysis of the samples were characterized by SEM (Phenom Pro) and TEM (Talos F200X G2). X-Ray diffraction measurements were performed using a Bruker X-Ray D8 Advance diffractometer using Cu Kα radiation (λ = 1.54 Å). UV-VIS-NIR Spectrometer (Lamda 750) was used to measure the absorption spectrum of the sample.

Photo-battery Design: First, 8 mm hole was machined on case of coin cell (CR2450) followed by sealing with a glass window using EVO epoxy. Then assembled the photo-batteries subsequently placing photocathode, Whatman glass microfiber filters paper separator, 200 μL of 3 M $Zn(CF_3SO_3)_2$ aqueous electrolyte, Zn (0.25 mm thick) anode, a stainless steel spacer (0.5 mm) with a spring followed by standard process.

Electrochemical Tests: CV tests of the photo-batteries were measured at different scan rates range from 0.2 mV s^{-1} to 10 mV s^{-1} over a voltage range of 0.2 V to 1.4 V using a Biologic VMP-3 galvanostatic battery cycler in dark and illuminated conditions. The galvanostatic discharge-charge tests were acquired at different specific currents range from 200 mA g^{-1} to 5000 mA g^{-1} in dark and illuminated conditions. AC impedance tests were measured (after the 2nd galvanostatic discharge cycle to 0.7 V) in a frequency range of 10 mHz - 100 kHz at a 10 mV amplitude both in dark and illuminated conditions. Furthermore, the photocharging measurements of the photo-batteries were tested by recording open circuit voltage under illumination and then discharged by applying specific currents.

***Ex-situ* UV-VIS Measurement:** To measure the *ex-situ* UV-VIS absorption, the photocathodes are discharged and charged at 500 mA g^{-1} to the SoC denoted in Figure S5a. The cycled electrodes were cleaned with deionized water and then dispersed in ethanol to measure the absorption spectra using PerkinElmer UV/Vis/NIR Spectrometer (Lamda 750).

Fabrication of Photodetectors and Electrical Measurements: The electrical photoresponse of VO_2 was measured by patterning Au/Cr (40/10 nm) IDEs on a Si_3N_4/Si wafer using UV lithography subsequently drop casting VO_2 on the IDEs to obtain planar Au-VO_2-Au photodetector. Current-voltage tests of Au-VO_2-Au photodetector are measured by sweeping the voltage from -0.1 V to +0.1 V in dark and illuminated conditions. Next, current-time measurements under alternative dark and illuminated conditions were recorded in absence (V = 0 V) and presence (V = 1 V) of an external bias voltage. Further, the FTO/ZnO/VO_2/Ag stacked photodetector was fabricated using layer-by-layer coating of ZnO and VO_2 on the FTO coated glass substrate subsequently drying at 120 ^{o}C in vacuum oven. Thereafter, Ag contacts were used to measure current-time test under alternative dark and illuminated conditions in absence of an external bias voltage (V = 0 V).

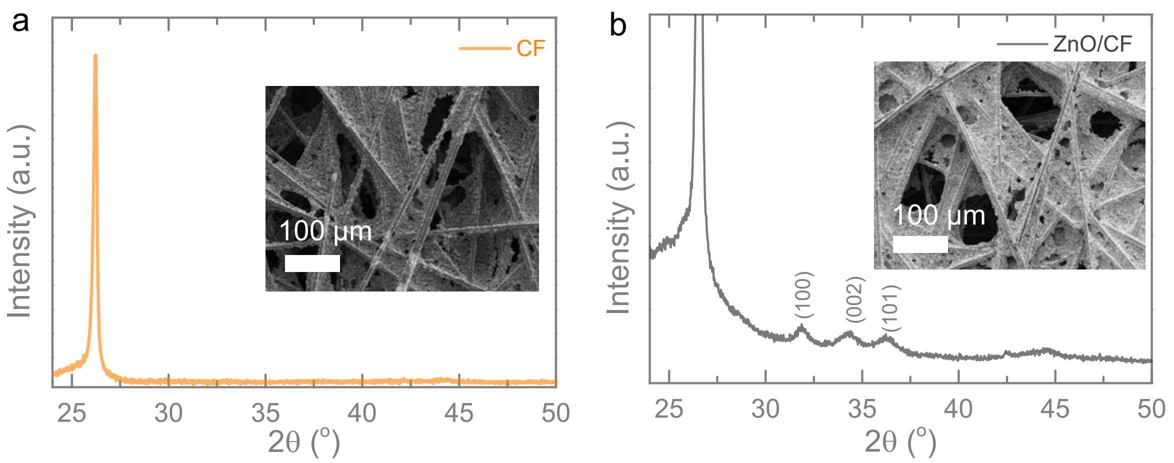

Figure S1. *XRD patterns (inset SEM images) of CF before and after coating of ZnO layer.*

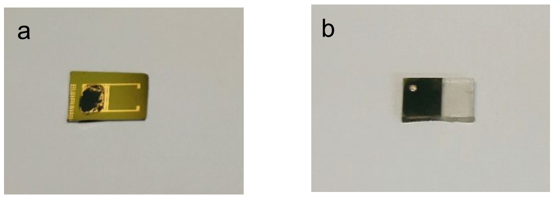

Figure S2. *Digital images of (a) Au-VO_2-Au and (b) FTO/ZnO/VO_2/Ag stacked photodetectors.*

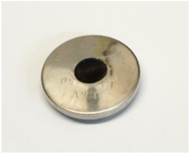

Figure S3. *Digital image of a photo-battery.*

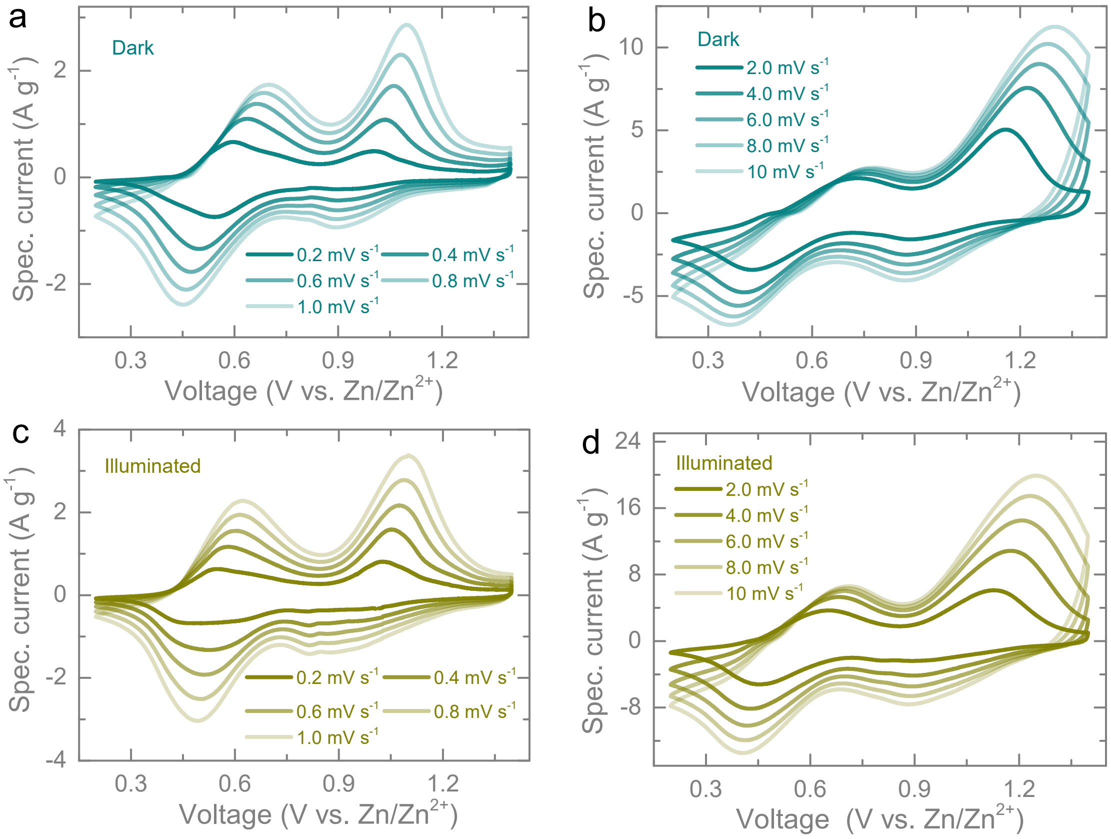

Figure S4. *CV curves at different scans of 0.2 mV s*$^{-1}$ *– 10 mV s*$^{-1}$ *in dark (a,b) and illuminated (c,d) conditions.*

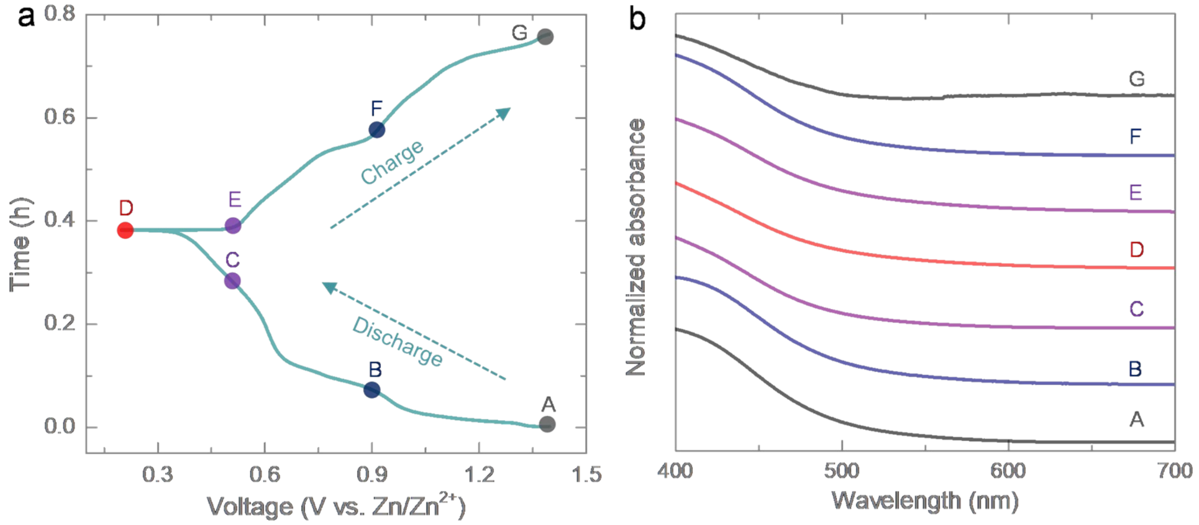

Figure S5. *(a) Second GDC cycle of a photo-battery. (b) Ex-situ absorbance spectra of the photocathodes at different SoC denoted in (a).*

Table S1. *Band gaps of VO_2 at different SoC denoted in Figure S5a.*

	A	B	C	D	E	F	G
Bandgap (eV)	2.38	2.38	2.40	2.41	2.36	2.39	2.38

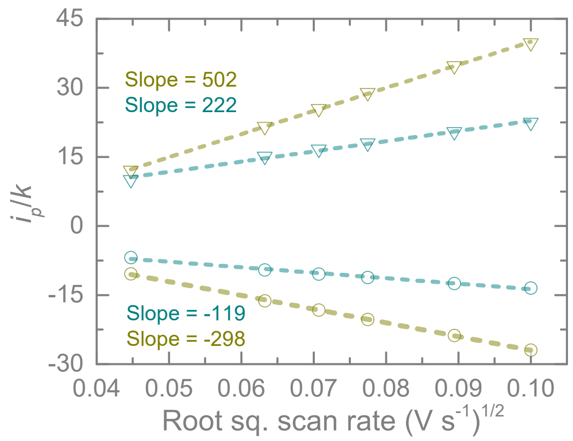

Figure S6. *Estimation of zinc ion diffusion constant improvements for major cathodic (C2) and anodic (A2) peaks in scan range of 2 mV s^{-1} – 10 mV s^{-2} under illumination.*

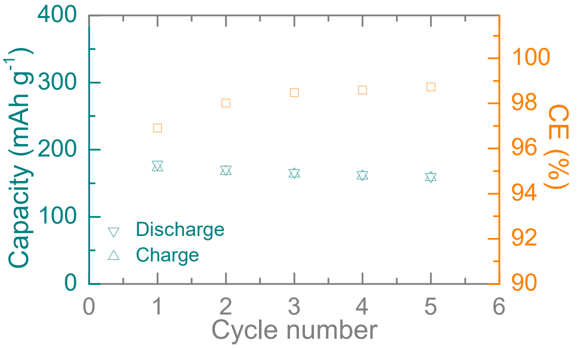

Figure S7. *Five formation cycles.*

Table S2. *Comparison of the photo-charging performance of this work with earlier photo-rechargeable energy storage reports.*

Photo-rechargeable energy storage system	Photo-charging voltage	Photo-conversion efficiency	Reference
g-C_3N_4//Zn capacitor	~ 850 mV	~0.01% under 420 nm illumination	*Nano Lett.*, 2020, **20**, 5967
AC//V_2O_5 zinc ion capacitor	~ 500 mV	~0.05% under 455 nm illumination	*ACS Energy Lett.*, 2020, **5**, 3132
V_2O_5//Li battery	~ 2.82 V	~2.6% for 455 nm illumination	*Nano Lett.*, 2021, **21**, 3527
V_2O_5//Zn battery	~ 950 mV	~1.2% under 455 nm illumination	*Energy Environ. Sci.*, 2020, **13**, 2414
2D perovskite//Li battery	~ 3.0 V	~ 0.034% under 420–650 nm illumination	*Nano Lett.*, 2018, **18**, 1856
$LiFePO_4$/N719-dye//Li battery	~ 3.65 V	~ 0.06–0.08% under 1 sun illumination	*Nat. Commun.*, 2017, **8**, 14643
VO_2//Zn battery	~ 890 mV	~ 0.18% under 455 nm illumination	*Adv. Energy Mater.*, 2021, **11**, 2100115
VO_2/ZnO//Zn battery	~ 880 mV	~ 0.51% for 455 nm illumination	This work

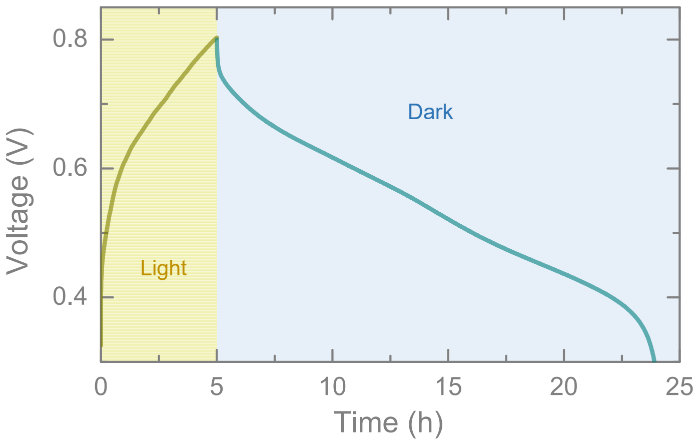

Figure S8. *Photocharge under 1 sun illumination of the photo-battery and galvanostatic discharge at a specific current of 0.02 mA cm^{-2}.*
